# Pediatric Trials Network: Stakeholder views on thanking families and providing study findings on pragmatic pediatric clinical research

**DOI:** 10.1016/j.conctc.2021.100792

**Published:** 2021-05-25

**Authors:** Amy Corneli, Brian Perry, Daniel K. Benjamin, Kanecia O. Zimmerman, Best Pharmaceuticals for Children Act – Pediatric Trials Network Steering Committee

**Affiliations:** a Duke Clinical Research Institute, Duke University, Durham, NC, USA; b Department of Population Health Sciences, Duke University, Durham, NC, USA; c Department of Pediatrics, Duke University, Durham, NC, USA

**Keywords:** Lay summary, Clinical trials, Pediatric trials network

## Abstract

We conducted formative research using in-depth interviews to identify preferences for and anticipated responses to receiving thank you notes and lay summaries of aggregate results among caregivers and adolescent participants of pragmatic pediatric studies conducted by the National Institute of Health-sponsored Pediatric Trials Network. We analyzed the data using qualitative thematic analysis. Nearly all participants said receiving a thank you note would make them feel valued, appreciated, and proud because they contributed to science. Similarly, nearly all participants said that receiving a lay summary of research results would make them aware of their role in improving the lives of children, feel like they are an active partner in research, and believe that researchers want to keep them informed. Participants also said that receiving a thank you note or lay summary may motivate them to participate in future research. Providing thank you notes as part of study participation should become a standard clinical trial practice, similar to the practice of providing lay summaries.

## Highlights

• Participants want to be thanked for their contributions to clinical research.
• Providing thank you notes and lay summaries demonstrate to caregivers and adolescent participants that researchers value and appreciate their contributions.
• Providing thank you notes and lay summaries may also increase caregivers’ and adolescent participants’ interest in participating in future research studies.

## 1 Introduction

As part of their patient and family engagement program, the National Institute of Health-sponsored Pediatric Trials Network (PTN) is dedicated to thanking participant families for their contributions to PTN studies and to sharing PTN study results with participants, their caregivers, and the Network at large. Sharing lay summaries [1] of aggregate research results with study participants has now become an expected norm in clinical research [2]. Several studies focused on pediatric research have reported on participants' and/or caregivers' interest in receiving aggregated research results and the type of information to include in summaries [[3], [4], [5]]. Reported less often are participants' reactions to receiving lay summaries of aggregated results of pragmatic pediatric clinical trials (i.e., clinical trials that are conducted in real-world settings). Additionally, while thank you notes have been used as a retention strategy in some clinical studies [6,7], limited evidence is available with regard to participants’ perspectives on receiving notes thanking them for their time and contributions to pragmatic pediatric clinical research.

We engaged two stakeholder groups of PTN studies—caregivers/parents and adolescent participants—in formative research on their preferences for and anticipated reactions to receiving thank you notes and aggregated lay summaries of pediatric clinical research results of pragmatic studies. Findings from this study allow stakeholder data to guide PTN decisions about how, when, where, and what to provide in thank you notes and lay summaries.

Here, we describe a subset of the study findings and focus on the perceived value of thank you notes and lay summaries as they are likely applicable to studies outside of PTN. Findings on the preferred content of thank you notes and lay summaries are presented elsewhere.

## 2 Methods

We conducted a qualitative descriptive study [8] with caregivers and adolescent participants of PTN studies using in-depth interviews. We aimed to enroll a diverse sample by interviewing adolescent participants and caregivers who were currently or previously enrolled in one of four PTN studies from multiple PTN sites across the United States (Table 1). PTN site staff assisted with recruitment by identifying and inviting caregivers of young children and adolescents who they believed may be interested in participating in this qualitative study to consider their participation, as well as participation for their child, when appropriate. Interested caregivers were connected with study staff, who were then linked with the adolescent participants.

**Table 1:** Participant characteristics.

Characteristic	No. (%) (n = 27)
Age	
14-17	3 (11.1)
18-24	1 (3.7)
25-34	10 (37.0)
35-44	10 (37.0)
45-49	3 (11.1)
Gender	
Female	27 (100)
Race^a^	
White	14 (51.9)
Black or African American	8 (29.6)
American Indian or Alaska Native	2 (7.4)
Asian	2 (7.4)
Other^b^	4 (14.8)
Ethnicity	
Hispanic or Latino	5 (18.5)
Current grade in school (adolescents, n = 3)	
8th—10th grade	3 (100)
Education (caregivers, n = 24)	
Some high school (9th to 12th grade)	1 (4.2)
High school diploma or equivalent	4 (16.7)
Some college credit	7 (29.1)
Associate degree	5 (20.8)
Bachelor's degree	6 (25.0)
Master's degree	1 (4.2)
Employment (caregivers, n = 24)	
Employed full-time	9 (37.5)
A homemaker	8 (33.3)
Employed part-time	4 (16.7)
Out of work and looking for work	1 (4.2)
A student	1 (4.2)
Unable to work	1 (4.2)
Location of affiliated PTN research sites	
Durham, North Carolina	6 (22.2)
Little Rock, Arkansas	5 (18.5)
Dallas, Texas	4 (14.8)
Wilmington, North Carolina	3 (11.1)
Jacksonville, Florida (Site 1)	3 (11.1)
Chicago, Illinois	3 (11.1)
Portland, Oregon	2 (7.4)
Jacksonville, Florida (Site 2)	1 (3.7)
Affiliated PTN study	
POP01^c^	13 (48.1)
AED01^d^	9 (33.3)
SCAMP/ABS01^e^	4 (14.8)
ANA01^f^	1 (3.7)

AED01, Pharmacokinetics of antiepileptics in obese children; ANA01, Pharmacokinetics and safety of anesthetics and analgesics in children and adolescents; POP01, Pharmacokinetics of Understudied Drugs Administered to Children Per Standard of Care; PTN, Pediatric Trials Network; SCAMP, Antibiotic Safety in Infants with Complicated Intra-abdominal Infections.

a Participants selected all that applied.

b Two participants indicated Hispanic.

c Study design: Opportunistic; Therapeutic Area: Several; Intervention: Multi-drug; Endpoint: PK.

d Study design: Opportunistic; Therapeutic Area: Seizures; Intervention: Levetiracetam, Valproic acid, Topiramate, Oxcarbazepine; Endpoint: PK and safety.

e Study design: Randomized; Therapeutic Area: Intra-abdominal infection in infants (<3 months); Intervention: ampicillin, metronidazole, clindamycin, piperacillin-tazobactam, gentamicin; Endpoint: Safety.

f Study design: Opportunistic; Therapeutic Area: Analgesia and Anesthesia; Intervention: Hydromorphone and Ketamine. Endpoint: PK/PD and safety.

During the one-on-one interviews, we asked participants to view several mock versions of thank you notes and lay summaries of PTN study results and describe their expectations of and anticipated reactions if they had received these materials as part of their PTN study participation. We also asked about a pre-identified list of potential reactions if not initially mentioned by the participant. We conducted the interviews by telephone, and they were audio-recorded with participant permission, transcribed, and analyzed using applied thematic analysis [9].

Using NVivo 12 [10], two analysts first segmented participants’ transcript text into structural codes (i.e., conceptual categories based on the topics explored in the interviews). Analysts conducted inter-coder reliability checks, discussed discrepancies, and revised transcript segmentation when necessary. Next, analysts inductively identified and applied content-derived codes to the text in each structural coding report, allowing for identification of issues salient to participants. The analysts then organized content codes thematically depending on the relationships between codes. Analysts produced analytical memos summarizing the main findings, together with illustrative quotes.

The Duke University Health System Institutional Review Board (IRB) reviewed and approved the study, waiving informed consent for the caregiver interviews. For adolescent interviews, adolescents provided their oral informed assent and their caregivers provided parental permission.

## 3 Results

### 3.1 Study participants

We interviewed 27 participants (24 caregivers and 3 adolescents) of diverse race, ethnicity, education level, employment, geographic location, and disease state (Table 1).

### 3.2 Thank you notes

Most participants said caregivers and adolescent participants should receive thank you notes because the notes demonstrate gratitude for their research contributions. Nearly all said receiving a thank you note would make them feel valued, appreciated, and proud because they contributed to science. Participants said:

*[Receiving a thank you note] would make me feel appreciated … like I made a difference in something.* —Adolescent, age 14

*[Receiving a thank you note] would give me a sense of purpose. You're not just a piece of data. You've got something else going on. You're involved.* —Caregiver, age 47

Some caregivers expressed that their efforts caring for children with severe illnesses are often unrecognized, and a thank you note would be a source of encouragement. A 26-year-old caregiver said:

*I think that [caregivers] should be thanked. It's hard on us being caregivers. We don't get to clock out. It's a daily struggle. It just feels good to know that there are people out there that support you. They feel for you when it comes to each individual situation.*

*[Caregivers] can be thanked, and maybe know that your child's research is very significant. It's just imperative that [caregivers] feel like they have contributed to something. We are truly grateful that we could help someone else's child. To know that the study helped prevent another child from going through the different adverse effects, that really means a lot to us.*

Some also felt that providing a thank you note may promote altruism and interest in participating in future studies, including among adolescents. A 39-year-old caregiver explained:

*As a parent of a child [with many health issues] … I think [receiving a thank you note] would encourage me to participate in more studies, to know that my kid helped someone find out information.*

Participant preferences varied on the timing of providing thank you notes, and some suggested the length of the trial should be considered. For example, one thank you note can be provided at the end of shorter trials and two notes for longer trials—one after enrollment and another at the end. Some participants cautioned on losing sentiment with providing too many thank you notes.

### 3.3 Lay summaries

Most participants said caregivers should receive a lay summary of the research results, and about one-third said adolescents should. Participants stressed the importance of informing caregivers of the results, and some explained that caregivers can decide if and when to inform their child. Nearly all said receiving a lay summary would make them aware of their role in improving the lives of children, feel like they are an active partner in research, believe that researchers want to keep them informed, and motivated to participate in future research. Participants said:

*[Receiving a lay summary] would make me feel good. Because a lot of times, when you [participate in] these studies in the past, I don't know what came of that. I think [getting a lay summary] gives you more closure than anything. If you actually have it printed out in front of you, and [even though] you're not named individually, you personally know how you answered questions … this is what I contributed to.* —Caregiver, age 45

*[I would] definitely [take part in other research] because they are involving you in the study. They are keeping you in the loop basically. And next time, you're like, “Okay, I'll enroll my child in other studies because they always keep me informed.”* —Caregiver, age 31

Most also expressed value in providing a lay summary years after their individual participation is over, acknowledging the long clinical trial timeline, and noted their expectation to receive individual-level results. A 37-year-old caregiver said:

*I'd be more likely [to take part in future research] if I knew the results of one study and was thanked. Knowing that the research team actually cared that you participated, and cared that you found out the results of the study, no matter how long it takes, [makes] you feel like your participation mattered.*

## 4 Discussion

Our findings provide new evidence suggesting that participants want to be thanked for their participation in research and value the acknowledgement. Our findings also contribute to existing evidence on the importance of lay research summaries by demonstrating the value and appreciation that participants place on receiving such information for pragmatic pediatric clinical research. Because of the anticipated participant response and ease of provision, we recommend that participant thank you notes become an expected study procedure in all clinical research similar to the provision of lay summaries.

Participants’ narratives also suggest that providing thank you notes and lay summaries may motivate future research participation. Research has shown that receiving a lay summary of research findings increases interest in participation in future research [11]; such data are not available on the provision of thank you notes. PTN is planning a follow-up study to assess the effect of receiving trial participation thank you notes and subsequent participation in pediatric research.

## Author contributions

Dr Corneli conceptualized and designed the study, designed the data collection instruments, drafted the initial manuscript, and reviewed and revised the manuscript.

Dr. Zimmerman conceptualized and designed the study, and critically reviewed the manuscript for important intellectual content.

Mr. Perry designed the study, coordinated and supervised data collection, collected data, carried out the analyses, and critically reviewed the manuscript for important intellectual content.

Dr. Benjamin conceptualized the study, obtained funding, and critically reviewed the manuscript for important intellectual content.

All authors approved the final manuscript as submitted and agree to be accountable for all aspects of the work.

## Conflict of interest disclosures

The authors have no conflicts to disclose.

## Funding/support

This work was funded under the 10.13039/100000071National Institute of Child Health and Human Development (NICHD) contract (HHSN275201000003I) for the Pediatric Trials Network (PI Danny Benjamin). The content is solely the responsibility of the authors and does not necessarily represent the official views of the National Institutes of Health.
