# Supplementary material for: Pediatric Trials Network: Stakeholder views on thanking families and providing study findings on pragmatic pediatric clinical research
Source: Contemp Clin Trials Commun. 2021 May 25;22:100792. doi: 10.1016/j.conctc.2021.100792 (PMC8181207; doi:10.1016/j.conctc.2021.100792)
Supplement: Multimedia component 1 [file mmc1.docx]

**Appendix**

**PTN Steering Committee Members:** Daniel K. Benjamin Jr., Christoph Hornik, Kanecia Zimmerman, Phyllis Kennel, and Rose Beci, Duke Clinical Research Institute, Durham, NC; Chi Dang Hornik, Duke University Medical Center, Durham, NC; Gregory L. Kearns, Scottsdale, AZ; Matthew Laughon, University of North Carolina at Chapel Hill, Chapel Hill, NC; Ian M. Paul, Penn State College of Medicine, Hershey, PA; Janice Sullivan, University of Louisville, Louisville, KY; Kelly Wade, Children's Hospital of Philadelphia, Philadelphia, PA; Paula Delmore, Wichita Medical Research and Education Foundation, Wichita, KS

**The Eunice Kennedy Shriver National Institute of Child Health and Human Development (NICHD):** Perdita Taylor-Zapata and June Lee

**The Emmes Company, LLC (Data Coordinating Center):** Ravinder Anand, Gaurav Sharma, Gina Simone, Kim Kaneshige, and Lawrence Taylor

**PTN Publications Committee:** Chaired by Thomas Green, Ann & Robert H. Lurie Children's Hospital of Chicago, Chicago, IL
